# Centripetal Axonal Transport as a Gateway to the CNS for Veterinary Antiparasitics: Bypassing the Blood–Brain Barrier, Clinical Impact in Vulnerable Age Groups, and the Potential Facilitating Role of PFAS

**DOI:** 10.1155/jt/1785323

**Published:** 2026-04-01

**Authors:** Domenico Britti, Romano Marabelli, Luigino Calzetta

**Affiliations:** ^1^ Department of Experimental and Clinical Medicine, University of Catanzaro, Catanzaro, Italy, unicz.it; ^2^ World Organization for Animal Health, Paris, 75017, France; ^3^ Department of Medicine and Surgery, Respiratory Disease and Lung Function Unit, University of Parma, Parma, Italy, unipr.it

**Keywords:** age-related susceptibility, antiparasitic drugs, blood–brain barrier, canine tremors, centripetal axonal transport, neurotoxicity, PFAS

## Abstract

The widespread use of veterinary antiparasitics, including neonicotinoids, isoxazolines, avermectins, and pyrethroids, is essential for canine health but raises concerns regarding potential neurotoxicity, particularly in young and geriatric animals. While the blood–brain barrier (BBB) offers significant protection, this review confronts a central “Safety Paradox”: how can a drug class with a demonstrated high therapeutic index in conventional safety studies be associated with a significant and persistent number of real‐world neurological adverse events? We propose that centripetal (retrograde) axonal transport represents a critical, underappreciated pathway for these compounds to access the central nervous system (CNS) from peripheral nerve terminals, offering a mechanistic solution to this paradox. This mechanism, well documented for various toxins and pathogens, could allow antiparasitics to bypass the BBB, leading to direct neuronal effects and contributing to clinical signs such as tremors. We review the neurotoxic mechanisms of these common antiparasitics, the established principles of retrograde axonal transport, and the chemical properties that make them candidates for such transport. Furthermore, we introduce the expanded hypothesis that per‐ and polyfluoroalkyl substances (PFASs)—present not only as “inert” ingredients or contaminants but, as recent regulatory findings reveal, also as active ingredients themselves—could act as both facilitators of transport for other neurotoxicants and as direct, cotransported neurotoxic agents. This review synthesizes existing and recent evidence to build a compelling case for axonal transport as a significant contributor to antiparasitic neurotoxicity, discusses its potential for differential clinical impact in young and old dogs, and highlights the urgent need for research into this pathway and the complex toxicological role of formulation components like PFAS.

## 1. Introduction

The “Safety Paradox” of veterinary antiparasitics and vulnerable populations: Effective parasite control is a cornerstone of canine health, heavily reliant on pharmacological interventions including neonicotinoids, isoxazolines, avermectins, and pyrethroids [1]. While these agents are generally considered safe at prescribed doses, a significant contradiction has emerged, creating a “Safety Paradox” that demands a new mechanistic explanation. On one hand, manufacturer‐led studies and regulatory documents emphasize a wide margin of safety for drugs like isoxazolines, often showing tolerability at multiples of the recommended dose (e.g., [2]). On the other hand, a growing body of independent evidence points to a notable incidence of real‐world neurological adverse events [3, 4].

This discrepancy is powerfully substantiated by recent clinical data. The U.S. Food and Drug Administration [5] first issued a safety communication in 2018 regarding neurological adverse events associated with the isoxazoline class, a warning that has been updated multiple times, including in 2019, indicating this is an ongoing regulatory concern. This persistent vigilance from the FDA demonstrates an ongoing regulatory concern and surveillance of this specific class of drugs for these particular adverse events. Furthermore, a 2024 retrospective analysis of UK and Dutch poison control center cases (2014–2023) confirmed that muscle tremors and convulsions are the most frequently reported neurological signs associated with isoxazolines [6]. The onset of these signs can be delayed up to 30 h, a pattern consistent with a transport‐based mechanism rather than immediate systemic toxicity. Underscoring the scale of the issue, the large‐scale “Project Jake” survey of pet owners and veterinarians reported that 66.6% of dogs administered an isoxazoline‐containing product experienced an adverse event, with muscle tremors and ataxia being commonly reported [7]. This highlights a potential discrepancy between official reporting and real‐world incidence, suggesting that adverse events may be more common than acknowledged and strengthening the need for alternative mechanistic explanations.

Limitations note: While the Project Jake survey provides valuable signal‐level evidence, its adverse‐event estimates may be influenced by self‐selection, recall bias, and variable case verification; therefore, mechanistic confirmation using quantitative transport assays and controlled exposure models is required.

The central nervous system (CNS) is largely protected by the blood–brain barrier (BBB), a complex interface that restricts the entry of many xenobiotics [8]. However, the BBB is not impervious; its integrity can vary with age and health status, and its protective capacity can be circumvented. These concerns are amplified when considering vulnerable populations, such as young dogs with developing nervous systems and geriatric dogs with age‐related physiological changes [9, 10].

This review posits a critical hypothesis that directly addresses the Safety Paradox: centripetal (retrograde) axonal transport serves as a significant, direct conduit for common veterinary antiparasitic substances to enter the CNS from peripheral nerve endings, thereby bypassing the conventional BBB. This mechanism, exploited by numerous biological toxins and environmental neurotoxicants [11, 12], could explain the occurrence of CNS‐related adverse effects, especially when direct BBB penetration seems limited, when multiple neuroactive compounds are coadministered, or in animals with heightened age‐related susceptibility. By providing an alternative “back door” pathway, this mechanism offers a mechanistic solution to the puzzling discrepancy between trial‐based safety and real‐world neurological adverse events.

Furthermore, we introduce an expanded subhypothesis: the presence of per‐ and polyfluoroalkyl substances (PFASs) in antiparasitic formulations, either as excipients, contaminants, or as active ingredients themselves, may facilitate, enhance, or act in an additive/synergistic manner to promote the neurotoxicity of these products.

This paper will (i) review the neurotoxic mechanisms of key veterinary antiparasitic classes, (ii) detail the established principles of centripetal axonal transport and its role in neurotoxicology, (iii) analyze the physicochemical properties of antiparasitics that predispose them to axonal transport, (iv) explore the redefined role of PFAS in modulating this transport pathway as facilitators and direct toxicants, and (v) discuss the potential for synergistic neurotoxicity, the clinical implications for vulnerable age groups, and future research directions.

## 2. Neurotoxic Mechanisms of Common Canine Antiparasitics

The primary classes of antiparasitics used in canine medicine each possess distinct mechanisms of action that, while selective for invertebrates, can have off‐target effects on the mammalian nervous system. A summary of these mechanisms is provided in Table 1.

**TABLE 1:** Summary of neurotoxic mechanisms of key veterinary antiparasitics.

Antiparasitic class	Example compounds	Primary molecular target(s)	Mechanism of action	Associated neurological signs
Neonicotinoids	Imidacloprid, nitenpyram	Nicotinic acetylcholine receptors (nAChRs)	Agonist; persistent neuronal stimulation	Tremors, hyperexcitability, lethargy
Isoxazolines	Fluralaner, afoxolaner	GABA‐ and glutamate‐gated chloride channels	Noncompetitive antagonist; inhibits Cl−influx	Tremors, ataxia, seizures
Avermectins	Moxidectin, ivermectin	GABA‐A receptors (mammals), GluCls (invertebrates)	Potentiates GABAergic inhibition	CNS depression, ataxia, tremors
Pyrethroids	Permethrin, deltamethrin	Voltage‐gated sodium channels	Prolongs Na+ influx; repetitive firing	Tremors, paresthesia, seizures

### 2.1. Neonicotinoids (e.g., Imidacloprid and Nitenpyram)

Neonicotinoids are agonists of nicotinic acetylcholine receptors (nAChRs), causing persistent neuronal stimulation [13]. While exhibiting selectivity for insect nAChRs, mammalian nAChRs can also be affected, particularly at higher concentrations or with prolonged exposure. Mammalian neurotoxicity can manifest as tremors, hyperexcitability, and lethargy, indicating CNS involvement [14].

### 2.2. Isoxazolines (e.g., Fluralaner, Afoxolaner, Lotilaner, and Sarolaner)

Isoxazolines act as non‐competitive antagonists of GABA‐gated chloride channels (GABACls) and glutamate‐gated chloride channels (GluCls) in invertebrates, inhibiting chloride influx and causing neuronal hyperexcitation [15]. While displaying selectivity for invertebrate channels, off‐target effects on mammalian GABA‐A receptors can occur, potentially leading to tremors, ataxia, and seizures by reducing GABAergic inhibition [4]. The regulatory‐mandated warning language now standard on all fluralaner‐containing products explicitly states that the isoxazoline class “has been associated with neurologic adverse reactions including tremors, ataxia, and seizures,” and, critically, that “seizures have been reported in dogs receiving isoxazoline class drugs, even in dogs without a prior seizure history” [2]. This explicit warning from regulatory bodies validates the clinical relevance of the neurological signs being investigated and underscores the ongoing concern.

A pivotal development is the recent approval (2023–2025) and marketing of BRAVECTO QUANTUM, an extended‐release injectable suspension of fluralaner designed to provide 12 months of continuous, systemic exposure from a single administration [16]. This formulation marks a fundamental shift in the toxicological exposure paradigm from acute or subchronic events to one of chronic, low‐level, and uninterrupted exposure. A recent European field study confirmed its year‐long efficacy and reported a favorable safety profile, with only transient tiredness and decreased appetite noted as related adverse events [17]. However, this new paradigm significantly strengthens the axonal transport hypothesis, as a slow, persistent “drip” of a neurotoxicant directly into neuronal cell bodies over a year could lead to a cumulative toxic burden that eventually surpasses a clinical threshold, a scenario not previously encountered with shorter‐acting products. This long‐term, low‐level exposure model is particularly conducive to the accumulation of toxicants via a mechanism like axonal transport, making the “back door” pathway even more clinically relevant.

### 2.3. Avermectins (e.g., Moxidectin, Ivermectin, and Selamectin)

Avermectins primarily potentiate GluCls in invertebrates, causing flaccid paralysis. In mammals, which lack CNS GluCls, avermectins can interact with GABA‐A receptors, potentiating chloride influx. This can lead to CNS depression, ataxia, and, paradoxically, tremors, particularly at toxic doses [18]. Their neurotoxicity in mammals is significantly modulated by P‐glycoprotein (P‐gp, encoded by the ABCB1/MDR1 gene) at the BBB, which actively effluxes these compounds from the CNS [19]. Dogs with MDR1 mutations are highly susceptible [20].

### 2.4. Pyrethroids (e.g., Permethrin and Deltamethrin)

Pyrethroids act by modifying the gating kinetics of voltage‐gated sodium channels, prolonging sodium influx and leading to repetitive neuronal firing and hyperexcitability [21]. Clinical signs of pyrethroid toxicity in mammals include tremors (often described as “paresthesia‐tremor” or “T‐syndrome”), ataxia, hypersalivation, and seizures [22]. Due to their lipophilicity, they can readily access nervous tissue.

## 3. Centripetal Axonal Transport: A “Back Door” to the CNS

### 3.1. The Physiological Pathway and Its Hijacking by Neurotoxicants

Retrograde axonal transport is a vital physiological process, utilizing dynein motor proteins along microtubule tracks to move organelles, signaling molecules, and waste products from the axon terminal to the neuronal soma [23]. This pathway maintains neuronal health and communication. However, its machinery can be co‐opted by various exogenous agents, providing a direct route into the nervous system that bypasses traditional barriers.

Well‐established examples of hijacking are as follows:

• **Biological toxins:** Tetanus toxin and botulinum neurotoxins are classic examples. They bind to peripheral nerve terminals, are internalized into vesicles, and are transported retrogradely to their sites of action within the CNS (for tetanus toxin) or motor neuron somata (for botulinum toxin), respectively [24].
• **Viruses:** Pathogenic viruses such as rabies virus [25] and herpes simplex virus [26] exploit this route for neuroinvasion and spread within the nervous system. Rabies virus, for instance, specifically targets the retrograde transport machinery to reach the CNS from peripheral infection sites.
• **Heavy metals:** Mercury [27, 28], manganese [29] (especially via the olfactory pathway which offers direct axonal entry to the brain), and lead [30] have all been shown to undergo retrograde axonal transport, contributing to their central neurotoxicity.
• **Industrial chemicals and pesticides:** The anticancer drug doxorubicin is transported retrogradely in peripheral nerves [31]. Acrylamide, known for causing “dying‐back” axonopathy, involves complex mechanisms including the disruption of axonal transport and potential retrograde signaling of damage or transport of the toxicant itself or its adducts [32]. Crucially, recent evidence confirms that organophosphate (OP) insecticides are known to cause defects in axonal transport by disrupting both kinesin motor protein function and the polymerization of tubulin [33]. This establishes a powerful precedent: axonal transport is a known, contemporary target for other major insecticide classes, making the hypothesis that veterinary antiparasitics utilize this same pathway far more plausible.

Furthermore, recent imaging innovations allow in vivo SPECT visualization of net retrograde transport, making direct testing of the present hypothesis technologically feasible [34]. This advancement is critical for moving beyond in vitro or ex vivo models to directly observe and quantify transport in living animals.

### 3.2. Physicochemical Properties Favoring Antiparasitic Axonal Transport

For antiparasitics to engage this pathway, they must reach peripheral nerve terminals (e.g., in the skin, neuromuscular junctions, and sensory endings in the gut), interact with or cross the axonal membrane, and become associated with retrograde transport carriers. Several chemical properties of common antiparasitics, summarized in Table 2, make them plausible candidates.

• Lipophilicity: Many pyrethroids, avermectins, and isoxazolines are moderately to highly lipophilic (e.g., fluralaner log P > 5) [35]. High lipophilicity aids in partitioning into the lipid‐rich neuronal membranes, facilitating entry into the axon terminal and potentially nonspecific incorporation into membrane‐bound vesicles destined for retrograde transport [36]. Given that axolemmal and vesicular membranes are lipid rich, higher log P values are expected to favor partitioning at peripheral nerve terminals, thereby increasing the likelihood of endocytic uptake and incorporation into retrogradely transported vesicles.
• **Molecular size:** Most of these insecticide molecules are relatively small organic compounds compared with large protein toxins. Their size generally does not preclude them from being endocytosed or from diffusing across membranes (if sufficiently lipophilic) and can be advantageous for “hitchhiking” on various transport vesicles [37, 38].
• **Receptor/channel interaction and trafficking:** All these insecticide classes bind to specific ion channels or receptors (nAChRs, GABA/GluCls, GABA‐A receptors, and sodium channels). These target proteins are often present on peripheral nerve terminals and are themselves subject to cellular trafficking processes, including endocytosis and subsequent transport. If the insecticide remains bound to its target during these processes, it could be carried along. For example, ligand‐bound receptors are frequently internalized for signaling resolution or degradation, and these endosomes can be sorted into retrograde pathways [39]. Recent work on the TrkB receptor, for instance, highlights the role of Rab10 as a critical component of its retrograde transport machinery, underscoring the specificity and complexity of these internal trafficking pathways [40].
• **Metabolic stability:** For a toxicant to exert an effect on the soma after retrograde transport, it must remain sufficiently intact or be converted to an active metabolite that is then transported. Many antiparasitics are designed for a degree of persistence to ensure efficacy.

**TABLE 2:** Physicochemical properties and transport precedent for antiparasitics and facilitators.

Compound/class	Representative compound(s)	Lipophilicity (log P)	Key property/precedent for axonal transport	Reference
Isoxazolines	Fluralaner	> 5	High lipophilicity favors membrane partitioning.	[35]
Avermectins	Ivermectin	∼4.5	Lipophilic; P‐gp substrate (relevant for BBB bypass).	[35]
Pyrethroids	Permethrin	∼6.5	Highly lipophilic; readily accesses nervous tissue.	[35]
PFAS	PFOA/PFOS	Varies	Amphiphilic; surfactant activity alters membrane permeability.	[41]
Organophosphates	(e.g., Chlorpyrifos)	∼4.7	Established precedent: known to disrupt axonal transport machinery.	[33]

### 3.3. Plausible Mechanisms of Antiparasitic Uptake and Transport

• **Receptor-mediated endocytosis:** Binding to surface receptors/channels followed by internalization.
• **Lipid raft/caveolae-mediated endocytosis:** Accumulation of lipophilic compounds in these specialized membrane microdomains, followed by endocytosis.
• **Fluid-phase endocytosis (pinocytosis):** Nonspecific uptake of extracellular fluid containing dissolved antiparasitics.
• **“Hitchhiking” on damaged organelles or signaling endosomes:** Localized neurotoxicity or stress at the terminal induced by the antiparasitic could trigger the retrograde transport of stress signals or damaged organelles (e.g., via autophagosomes), with the antiparasitic associated with these structures. Antiparasitics might also partition into the membranes of normally trafficking signaling endosomes. For example, brain‐derived neurotrophic factor (BDNF) is known to be transported retrogradely in signaling endosomes, providing a sophisticated molecular mechanism that could be hijacked [42].

**FIGURE 1:**
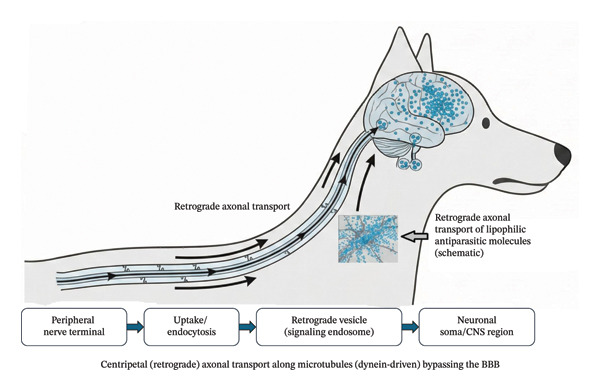
Hypothesized pathway of centripetal (retrograde) axonal transport of veterinary antiparasitic compounds from peripheral nerve terminals to the canine central nervous system (CNS), bypassing the blood–brain barrier (BBB).

These proposed uptake mechanisms converge on a common pathway of centripetal (retrograde) axonal transport toward neuronal somata, as schematically illustrated in Figure 1.

Lipophilic antiparasitic molecules present at the skin–nerve interface may undergo uptake at peripheral nerve terminals via endocytic processes and be incorporated into retrogradely transported vesicles (signaling endosomes). These vesicles are conveyed along axonal microtubules toward neuronal somata through dynein‐dependent transport, potentially enabling the cumulative exposure of central neuronal structures independently of passive BBB penetration.

## 4. The Redefined Role of PFAS: From “Inert Facilitator” to “Active and Additive Toxicant”

Terminology note: In this review, “inert PFAS” refers to PFAS present as excipients (e.g., surfactants and dispersants) or contaminants (including packaging‐related sources), whereas “active‐ingredient PFAS” refers to fluorinated organic active substances that meet broad structural PFAS definitions (e.g., as identified in the 2025 Minnesota Department of Agriculture report). This distinction is essential for evaluating facilitation, direct transport, and additive/synergistic risk.

The original hypothesis cast PFASs as “inert” ingredients or contaminants that could facilitate the axonal transport of active compounds. However, recent landmark regulatory findings compel a fundamental expansion of this hypothesis. The final 2025 report from the Minnesota Department of Agriculture (MDA) revealed that under the state’s broad, structure‐based definition, 97 pesticide ACTIVE ingredients registered for use are themselves classified as PFAS [43]. This discovery transforms the role of PFAS from a potential facilitator to a direct, and potentially additive, neurotoxicant. The American Veterinary Medical Association (AVMA) now formally acknowledges the growing threat of PFAS to animals, referencing reports of PFAS detected in flea‐control products [44].

### 4.1. Relevant Properties of PFAS for Neuronal Interaction and Transport

• **Amphiphilicity and surfactant activity:** Many PFAS (e.g., PFOA and PFOS) possess both hydrophobic fluorinated tails and hydrophilic head groups, enabling them to act as surfactants [41]. Surfactants are known to alter cell membrane permeability and integrity, potentially facilitating the entry of other molecules [45].
• **Lipophilicity and membrane interaction:** The hydrophobic fluorinated tail allows strong interaction with lipid membranes, potentially leading to accumulation in axon terminals and incorporation into vesicular membranes [46].
• **Protein binding:** PFAS bind avidly to various proteins, including serum albumin, but also potentially to cellular proteins, including those on cell surfaces or within transport vesicles [47].
• **Persistence and bioaccumulation:** Their extreme resistance to degradation means that if PFAS reach nerve terminals, they may reside there for extended periods, increasing the opportunity for uptake into retrograde transport pathways or for chronic membrane interaction [48].
• **Intrinsic neurotoxicity:** A growing body of evidence indicates that some PFAS are neurotoxicants, particularly affecting neurodevelopment, and can accumulate in brain tissue although the mechanisms of their own CNS entry are still being fully elucidated [49]. These intrinsic neurotoxic effects include oxidative stress, mitochondrial dysfunction, disruption of calcium homeostasis, and altered neurotransmitter systems, further compounding the potential for harm.

### 4.2. A Multipronged Hypothesis for PFAS‐Related Neurotoxic Risk

The new evidence supports a more comprehensive, multipronged model for PFAS‐related neurotoxic risk.

• **The facilitation model (original hypothesis):** Inert PFAS, present as surfactants or contaminants from packaging, may increase the permeability of axonal membranes or, otherwise, enhance the uptake and transport of coformulated, non‐PFAS active ingredients like isoxazolines. Concerns over packaging as a source are validated by the EPA’s development of a new method to detect PFAS in HDPE containers and its subsequent regulatory action under the Toxic Substances Control Act (TSCA) to address this contamination route [50].
• **The direct transport model (new hypothesis):** Active‐ingredient PFAS may themselves be candidates for centripetal axonal transport due to their own physicochemical properties. In this model, the PFAS is not a facilitator but is the primary neurotoxicant being delivered directly to the neuronal soma.
• **The synergistic/additive model (new hypothesis):** This model considers the reality of complex mixtures. Coformulations could lead to multiple neurotoxic interactions, such as an inert PFAS enhancing the transport of an active‐ingredient PFAS, or multiple neurotoxicants being transported simultaneously, creating an additive or synergistic toxic burden within the neuron. If PFASs reach the soma, their intrinsic neurotoxic effects could sensitize the neuron, making it more vulnerable to the effects of coexposed antiparasitics that arrive via any route (direct BBB penetration or separate axonal transport). This creates an additive or synergistic neurotoxic burden [51].

## 5. Synergistic Neurotoxicity, Clinical Implications, and Age‐Related Susceptibility

The potential for multiple neuroactive compounds to interact, coupled with the proposed axonal transport mechanism (potentially enhanced by PFAS), raises significant concerns for cumulative neurotoxicity.

### 5.1. Pharmacodynamic and Pharmacokinetic Interactions

• **Pharmacodynamic synergism/additivity:** Multiple antiparasitics acting on different neuronal targets (e.g., isoxazolines on GABA receptors, pyrethroids on sodium channels, and neonicotinoids on nAChRs) can converge to produce an overall increase in neuronal hyperexcitability, lowering the threshold for clinical signs like tremors.
• **Pharmacokinetic interactions:** Competition for metabolic enzymes (e.g., cytochrome P450s) can alter clearance rates [1]. Interactions at the BBB involving P‐glycoprotein are particularly relevant. Avermectins are well‐known P‐gp substrates [19]. Some isoxazolines (e.g., fluralaner) are also P‐gp substrates and may weakly inhibit P‐gp [52]. Such interactions can increase direct CNS penetration of P‐gp substrates. Critically, centripetal axonal transport bypasses the P‐gp efflux mechanism at the BBB endothelium although P‐gp or other transporters might be present on neuronal membranes themselves, influencing local uptake or retention at the terminal.

### 5.2. Clinical Manifestations (Tremors) and Age‐Related Susceptibility

Tremors are a common neurological sign associated with exposure to several of these antiparasitic classes. The proposed mechanisms could lead to an increased toxicant load in CNS motor control pathways. This risk is not uniform across the canine population.

#### 5.2.1. Young Dogs

The developing nervous system in puppies is particularly vulnerable.

• **Immature BBB:** The BBB is not fully mature at birth and continues to develop postnatally, potentially allowing greater passive diffusion of xenobiotics [53].
• **Dynamic axonal processes:** Axonal growth, synaptogenesis, and pruning are highly active, possibly leading to increased exposure of nerve terminals and more dynamic axonal transport rates [54].
• **Immature metabolism:** Detoxification pathways (e.g., hepatic enzymes) are often not fully developed in neonates and young animals, leading to slower clearance and prolonged exposure to parent compounds or active metabolites [55].
• **Heightened sensitivity:** Developing neurons may exhibit greater sensitivity to excitotoxic or disruptive effects of neurotoxicants [10].

#### 5.2.2. Geriatric Dogs

The aging nervous system presents a different set of vulnerabilities.

• **Increased BBB permeability:** Age‐related decline in BBB integrity (“leaky BBB”) has been documented, potentially allowing increased entry of systemic toxicants [56].
• **Reduced efflux capacity:** The efficiency of P‐gp and other efflux transporters at the BBB may decline with age, reducing the brain’s ability to clear xenobiotics [57].
• **Altered axonal transport:** Axonal transport mechanisms can become less efficient or more prone to disruption in aging neurons [58].
• **Diminished neuroprotection and repair:** The capacity for neuroprotection, neurogenesis, and repair diminishes with age, making neurons less resilient to toxic insults [9].
• **Comorbidities and polypharmacy:** Older dogs are more likely to have concurrent health issues and be on multiple medications, increasing the risk of drug interactions.

#### 5.2.3. Genetic Factors

• **MDR1 (ABCB1) gene mutation:** Dogs with this mutation have dysfunctional P‐gp, making them highly susceptible to avermectin neurotoxicity via direct BBB passage and potentially increasing the relative importance of any CNS entry via axonal transport for other P‐gp substrate antiparasitics [20].
• **Formulation effects:** The presence of PFAS or other unlisted excipients could represent an unappreciated susceptibility factor that disproportionately affects vulnerable age groups or individuals.

## 6. Discussion: Bridging the Gaps—From Periphery to Central Dysfunction and the “Inert” Ingredient Question

The hypothesis that centripetal axonal transport serves as a direct route for veterinary antiparasitics to the CNS, potentially facilitated or compounded by PFAS, offers a unifying framework to understand puzzling neurotoxic events, especially those occurring in young or geriatric animals, or with polypharmacy. This pathway could explain the following:

• CNS signs when direct BBB penetration by the active ingredient is presumed to be limited by P‐gp or rapid systemic metabolism.
• Selective vulnerability of certain neuronal populations if their peripheral terminals are particularly adept at uptake or if their axonal transport rates differ.
• The potential for “silent” accumulation of toxicants in the CNS over time with repeated exposures, leading to delayed or chronic neurotoxicity.
• Variability in individual responses that cannot be solely explained by active ingredient pharmacokinetics based on systemic circulation alone.

A critical aspect of this hypothesis is the role of formulation excipients. The “inert” ingredients in pesticide formulations are often proprietary, and their individual or combined toxicological profiles, especially concerning neurotoxicity or their ability to modulate the transport and bioavailability of active ingredients, are frequently understudied [59]. The potential for PFAS to act as facilitators of axonal transport, or as additive neurotoxicants, is particularly concerning given their persistence, bioaccumulative potential, and known biological activities, including neurodevelopmental effects [60]. Regulatory scrutiny of these “inerts” is essential.

While compelling, this hypothesis requires direct experimental validation. Such studies are technically demanding. However, the development of novel in vivo imaging techniques, such as using single‐photon emission computed tomography (SPECT) to quantify the net retrograde axonal transport of radiolabeled molecules in living animal models, now makes this hypothesis directly testable [34].

## 7. Conclusions and Future Directions: A Call for Re‐Evaluation and Research

Centripetal axonal transport is a biologically plausible and well‐documented mechanism for various neurotoxicants to bypass the BBB and access the CNS. The physicochemical properties of common veterinary antiparasitics (neonicotinoids, isoxazolines, avermectins, and pyrethroids) are consistent with their potential engagement of this pathway. The hypothesis that axonal transport contributes significantly to the CNS accumulation and subsequent neurotoxicity (e.g., tremors) of these antiparasitics in canines warrants urgent and rigorous investigation. This route could explain adverse events that are otherwise difficult to reconcile with known BBB permeability and P‐gp efflux, particularly in cases of polypharmacy or in genetically or age‐susceptible individuals.

The presence of PFAS in antiparasitic formulations as “inert” ingredients or contaminants introduces a critical, novel dimension to this hypothesis. Given their surfactant properties, membrane interactivity, and protein‐binding capacity, PFAS could plausibly enhance the uptake of active parasiticidal ingredients into peripheral nerve terminals and/or facilitate their subsequent centripetal axonal transport. Alternatively, or additionally, PFAS transported to the soma could exert their own neurotoxic effects, sensitizing neurons to the primary antiparasitics.

The clinical impact of CNS entry via axonal transport (potentially facilitated by PFAS) may be particularly pronounced and diagnostically challenging in vulnerable canine populations, specifically young dogs with developing nervous systems and geriatric dogs with age‐related neurophysiological decline. These age groups may exhibit heightened susceptibility due to factors including immature or compromised BBB function, altered metabolic capacities, and different dynamics of axonal transport and neuronal resilience.

Current regulatory frameworks for pesticide and veterinary drug safety may neither adequately consider the potential for centripetal axonal transport of active ingredients or the modulatory/additive neurotoxic role of formulation excipients like PFAS nor sufficiently account for age‐related vulnerabilities. A re‐evaluation of risk assessment paradigms is needed to incorporate these complex entry routes, interactions, and life‐stage sensitivities. Critically, the 2025 MDA report exposes a significant regulatory gap between broad, state‐level definitions of PFAS and narrower federal definitions. This discrepancy may be causing regulators to overlook significant sources of PFAS exposure and potential neurotoxic risk.

Future research imperatives are as follows:

• **Direct tracing studies:** Utilize the validated SPECT imaging technique [34] to directly measure the accumulation of radiolabeled fluralaner in the CNS of canine models following administration of the 12‐month injectable formulation [17]. This would involve in vivo animal models (including young and aged dogs, and MDR1‐genotyped animals) to directly visualize and quantify uptake at peripheral terminals and retrograde transport to specific CNS somal populations.
• **Formulation analysis:** Employ the EPA’s validated 2024 analytical method for detecting PFAS in HDPE [50] to conduct a comprehensive, independent analysis of commercial veterinary antiparasitic formulations to systematically identify and quantify both intentionally added and contaminant PFAS, guided by the classifications in the 2025 MDA report [43]. This will clarify the “inert” ingredient question.
• **Mechanistic synergy studies:** Use in vitro compartmentalized neuronal culture systems (e.g., Campenot chambers and microfluidic devices using canine‐derived or relevant neuronal cell lines) to test the hypothesis that coexposure to an isoxazoline and specific PFAS can enhance the rate of retrograde transport or lower the threshold for neuronal hyperexcitability.
• **Combined exposure and age-**specific in vivo models: Develop and utilize in vivo canine models (or appropriate surrogates) that incorporate realistic combined exposures to multiple antiparasitics and defined PFAS, specifically assessing neurological endpoints (behavioral, electrophysiological, and neuropathological) in young, adult, and geriatric animals.
• **Susceptibility biomarker identification:** Identify genetic (beyond MDR1), epigenetic, or physiological biomarkers that may predispose individual dogs, or specific age groups, to enhanced axonal transport or increased vulnerability to transported neurotoxicants.
• **Pharmacovigilance data mining:** Re‐examine existing veterinary adverse event–reporting databases using advanced analytical methods to identify patterns suggestive of axonal transport mechanisms (e.g., delayed onset and specific symptom profiles), formulation‐related effects, or disproportionate reporting in young or geriatric animals.
• **Regulatory science advancement:** Encourage dialog between researchers, regulators, and industry to develop testing guidelines and risk‐assessment frameworks that explicitly address alternative CNS entry routes like axonal transport and the toxicological impact of “inert” formulation ingredients, especially for vulnerable populations. Advocate for federal regulatory bodies (FDA and EPA) to harmonize their definitions of PFAS with broader, structure‐based classifications like those adopted by Minnesota to ensure a more comprehensive assessment of neurotoxic risk from complex chemical mixtures in veterinary drugs.

Addressing these research questions is paramount for a comprehensive understanding of antiparasitic neurotoxicity and for ensuring the continued safe use of these vital veterinary medicines, particularly in the most vulnerable canine populations. The “back door” to the CNS may be more significant than previously appreciated, with distinct implications across the lifespan that demand thorough investigation.

Practical implications for veterinary practice include heightened vigilance in young, geriatric, and genetically susceptible dogs (e.g., MDR1/ABCB1 variants), careful consideration of cumulative exposures (including long‐acting formulations and polypharmacy), and transparent discussion of neurological warning signs with owners.

## Funding

This research received no external funding.

Open access publishing was facilitated by Universita degli Studi Magna Graecia di Catanzaro, as part of the Wiley–CRUI‐CARE agreement.

## Ethics Statement

This article is a review of existing literature and does not involve any new studies with human participants or animals conducted by the authors.

## Conflicts of Interest

The authors declare no conflicts of interest.

## Data Availability

Data sharing is not applicable to this article as no new data were created or analyzed in this study. All information synthesized in this review is derived from publicly available sources, which are fully cited in the reference list.
